# GALAD score as a prognostic model for recurrence of hepatocellular carcinoma after local ablation

**DOI:** 10.1007/s00432-024-05760-z

**Published:** 2024-05-07

**Authors:** Wenying Qiao, Jiashuo Li, Yiqi Xiong, Jiasheng Zheng, Ronghua Jin, Caixia Hu

**Affiliations:** 1 Interventional Therapy Center for Oncology, Beijing You’an Hospital, Capital Medical University, 8 Xitoutiao, Youanmenwai Street, Fengtai District, Beijing, People’s Republic of China; 2 Beijing Di’tan Hospital, Capital Medical University, 8 Jingshun East Street, Chaoyang District, Beijing, People’s Republic of China; 3 Changping Laboratory, Beijing, People’s Republic of China

**Keywords:** Hepatocellular carcinoma, GALAD, Recurrence, Nomogram, Local ablation

## Abstract

**Background:**

Currently, the high recurrence rate still forms severe challenges in hepatocellular carcinoma (HCC) treatment. The GALAD score, including age, gender, alpha-fetoprotein (AFP), lens culinaris agglutinin-reactive AFP (AFP-L3), and des-gamma-carboxyprothrombin (DCP) was developed as a diagnostic model. However, evidence is still lacking to confirm the capability of the GALAD score to predict the recurrence of HCC.

**Methods:**

This study included 390 HCC patients after local ablation at Beijing You'an Hospital from January 1, 2018, to December 31, 2022. Firstly, the area under the receiver operating characteristic (ROC) curve (AUC) was calculated to assess the predictive capability of the GALAD score. Then, the Kaplan–Meier (KM) curve and log-rank test were used to compare the prognosis between two groups classified by GALAD score. Finally, a nomogram for high-risk patients was established by Lasso-Cox regression. It was assessed by ROC curves, calibration curves, and decision curve analysis (DCA).

**Results:**

The ROC curve (AUC: 0.749) and KM curve showed the GALAD score had good predictive ability and could clearly stratify patients into two groups through the risk of recurrence. Prognostic factors selected by Lasso-Cox regression contained tumor number, tumor size, and globulin. The nomogram for high-risk patients showed reliable discrimination, calibration, and clinical utility.

**Conclusion:**

This research displayed that the GALAD score is an effective model for predicting the recurrence of HCC. Meanwhile, we found the poor prognosis of the high-risk group and created a nomogram for these patients.

**Supplementary Information:**

The online version contains supplementary material available at 10.1007/s00432-024-05760-z.

## Introduction

Hepatocellular carcinoma (HCC) represents the sixth most prevalent cancer and the third leading cause of cancer-related death worldwide. Approximately 906,000 new HCC cases, as well as an estimated 830,000 HCC deaths, occurred in 2020 (Sung et al. 2021). The incidence of HCC is most prevalent in Asia, particularly in China, which accounts for nearly half of the global cases (Zou et al. 2022). As for patients with early-stage HCC, local ablation is served as an effective treatment, characterized by its minimal trauma and fewer complications (Chen et al. 2020; Weinstein and Ahmed 2018). Despite advances in ablation techniques these years, the prognosis for HCC patients remains unsatisfactory due to the high recurrence rate (Zheng et al. 2020; Shin et al. 2021). Therefore, it is especially vital to find appropriate methods for predicting the prognosis of HCC patients after local ablation.

For these years, Clinical biomarkers like alpha-fetoprotein (AFP), lens culinaris agglutinin-reactive AFP (AFP-L3), and des-gamma-carboxyprothrombin (DCP) have been extensively utilized for the screening, diagnosis, and prognosis of HCC (Chen and Sharma 2020; Song et al. 2016). However, the inadequate detection of HCC, primarily due to the low sensitivity of these biomarkers, remains a major factor for poor prognosis (Tayob et al. 2023). Other methods in the guidelines, such as the abdominal ultrasound (US), also come with various limitations (Galle et al. 2018). It includes the reliance on operator ability and the technical difficulties in obese patients or those with non-alcoholic fatty liver disease (Yang et al. 2019).

In order to improve the detection capability of HCC, a variety of models and scores are under exploration. The GALAD score is based on demographic parameters, including age, gender, and clinical biomarkers such as AFP, AFP-L3, and DCP (Johnson et al. 2014). Since the development of the GALAD score, many studies focused on its capability in the diagnosis of HCC. The outcomes of these researches confirmed that the combination of clinical characteristics demonstrated superior efficacy in the detection of HCC compared to using a single biomarker (Singal et al. 2022; Guan et al. 2023). Nevertheless, there is still an absence of evidence to verify the ability of GALAD score to forecast the prognosis of HCC.

Thus, a comprehensive study is required to validate the clinical significance of GALAD score, especially in prognosis prediction. The primary purpose of our study was to evaluate the effectiveness of the GALAD in predicting the recurrence of HCC patients and develop a nomogram using independent prognostic factors in order to provide guidance for clinicians.

## Methods

### Patients

This study enrolled 390 HCC patients who received ablation treatment at Beijing You’an Hospital, affiliated with Capital Medical University, between January 1, 2018, and December 31, 2022. The diagnosis of HCC was according to the American Association for the Study of Liver Disease (AASLD) guideline, which was based on the results of imaging examination or histologic diagnosis confirmed by liver biopsy (Marrero et al. 2018).

The inclusion criteria were as follows: (1) age from 18 to 75 years; (2) complete clinical and follow-up data; (3) Barcelona Clinic Liver Cancer (BCLC) stage 0 or A; (4) Child–Pugh class A or B. Patients who met the following exclusion criteria were considered ineligible: (1) received other treatment before ablation; (2) distant metastasis of HCC; (3) secondary liver cancer; (4) coagulation function disorders or serious diseases of vital organs, such as the heart, brain, lung, and kidney.

Approval for this research was obtained from the Ethics Committee of Beijing You'an Hospital, affiliated with Capital Medical University, ensuring adherence to the Declaration of Helsinki guidelines. The requirement for informed consent was waived due to the retrospective nature of this study (ethics approval number: 2024–083).

### Clinical characteristics and calculation of the GALAD score

Clinical characteristics were gathered during the seven days before the ablation, including (1) demographic data: age, gender, history of smoking, drinking, antiviral therapy, hypertension, and diabetes mellitus; (2) tumor information: tumor number (TN), tumor size (TS), BCLC stage, alpha-fetoprotein (AFP), lens culinaris agglutinin-reactive AFP (AFP-L3), and des-gamma-carboxyprothrombin (DCP). (3) liver function indicators: Child–Pugh class, liver cirrhosis, alanine aminotransferase (ALT), aspartate transaminase (AST), total bilirubin (TBIL), direct bilirubin (DBIL), albumin (ALB), and globulin (Glob). (4) other characteristics: prothrombin time (PT), activated partial thromboplastin time (APTT), international normalized ratio (INR), red blood cell (RBC), white blood cell (WBC), and hemoglobin (Hb).

GALAD score (gender, age, AFP, AFP-L3, and DCP) was included in this study to forecast the recurrence of HCC patients after local ablation (Johnson et al. 2014). The formula of GALAD is as follows: − 10.08 + 1.67 × gender (1 for males, 0 for females) + 0.09 × age + 0.04 × AFP-L3% + 2.34 × log_10_AFP + 1.33 × log_10_DCP.

### Treatment procedures

Local ablation treatments, such as radiofrequency ablation (RFA), microwave ablation (MWA), or argon-helium cryoablation (AHC), were performed by qualified hepatologists and interventional radiologists. Firstly, under the guidance of computed tomography (CT) or magnetic resonance imaging (MRI), the position and modality for ablation were determined. Subsequently, anesthesia was injected at the puncture site, and the ablation needle was inserted. In order to achieve complete ablation, multiple sites, overlapping, or repeated ablation should be considered, and the ablation area was extended 0.5–1.0 cm to ensure complete coverage. At last, the needle track was heated to prevent cancer implantation and postoperative bleeding. All patients underwent imaging examinations immediately after ablation, with the objective of assessing treatment efficacy.

### Follow-up

Patients with HCC were recommended to undergo regular follow-ups in line with clinical guidelines after local ablation. Typically, these patients were followed up for 3–6 months, which comprised clinicopathologic characteristics and adverse events. The last day of follow-up was July 1, 2023. Recurrence-free survival (RFS), the primary endpoint of this study, was defined as the time from local ablation to the date of first recurrence, last follow-up, or death.

### Statistical analysis

Categorical variables were expressed as frequencies (percentages), whereas continuous variables were reported as means ± standard deviation or medians (quartiles). Comparisons between two groups were analyzed using Student’s *t* test, Chi-square test, or non-parametric test as appropriate.

The receiver operating characteristic curve (ROC) was plotted, and the area under the ROC curve (AUC) was calculated to assess the performance of GALAD score and other GALAD biomarkers in HCC recurrence. The optimal GALAD score cut-off for predicting the recurrence of HCC patients in our cohort was reckoned according to the Youden Index, and it could divide patients into either a low-risk group or a high-risk group. Additionally, Kaplan–Meier (KM) curves and log-rank tests were used to compare the prognosis between two groups.

Lasso and Cox regression were performed to screen the independent prognostic factors, and a nomogram was developed for high-risk patients divided through GALAD score. ROC curves and AUCs were employed to evaluate the discrimination. Furthermore, the calibration and decision curve analysis (DCA) were used to validate the calibration performance and clinical utility. Patients were classified into low-risk, intermediate-risk, and high-risk groups on the basis of the nomogram. KM curves and log-rank tests were applied to compare the RFS between three groups.

All statistical analyses were conducted using R software (version 4.1.2). All tests were two-tailed, and statistical significance was set at P < 0.05.

## Results

### Baseline characteristics

In our retrospective cohort, 390 HCC patients who underwent ablation therapy from January 1, 2018, to December 31, 2022, at Beijing You’an Hospital affiliated with Capital Medical University were included. The median follow-up time was 29.9 months. As of the follow-up date, there were 181 cases of recurrence.

Patients’ baseline clinical characteristics were exhibited in Table 1. The average age of participants was 56.92 ± 9.16 years, including 299 (76.7%) males and 91 (23.3%) females. Among these 390 patients, 119 (30.5%) were diagnosed with hypertension, 95 (24.4%) with diabetes mellitus, and 357 (91.5%) with cirrhosis. 231 (59.2%) received antiviral treatment, and these patients were all infected with hepatitis B virus (HBV). Additionally, 165 (42.3%) had a history of smoking, and 126 (32.3%) had a history of drinking. Most patients were classified as Child–Pugh A (*n* = 299, 76.7%) and BCLC stage A (*n* = 267, 68.4%). In terms of tumor information, most tumors were single (*n* = 278, 71.3%) and smaller than 3 cm in size (*n* = 266, 68.2%). The median number of AFP was 18.53 ng/mL, while that of DCP was 49.00 ng/mL.

**Table 1:** Patient characteristics at baseline

Characteristic	All patients (*n* = 390)	Low-risk patients (*n* = 157)	High-risk patients (*n* = 233)	P value
Age	56.92 ± 9.16	54.99 ± 9.21	58.22 ± 8.92	**0.001**
*Gender* (%)				** < 0.001**
Male	299 (76.7)	138 (87.9)	161 (69.1)	
Female	91 (23.3)	19 (12.1)	72 (30.9)	
*Hypertension* (%)				0.98
Yes	119 (30.5)	48 (30.6)	71 (30.5)	
No	271 (69.5)	109 (69.4)	162 (69.5)	
*Diabetes* (%)				0.59
Yes	95 (24.4)	36 (22.9)	59 (25.3)	
No	295 (75.6)	121 (77.1)	174 (74.7)	
*Antiviral* (%)				0.06
Yes	231 (59.2)	102 (65.0)	129 (55.4)	
No	159 (40.8)	55 (35.0)	104 (44.6)	
*Smoking* (%)				0.59
Yes	165 (42.3)	69 (43.9)	96 (41.2)	
No	225 (57.7)	88 (56.1)	137 (58.8)	
*Drinking* (%)				0.07
Yes	126 (32.3)	59 (37.6)	67 (28.8)	
No	264 (67.7)	98 (62.4)	166 (71.2)	
*Cirrhosis* (%)				0.79
Yes	357 (91.5)	143 (91.1)	214 (91.8)	
No	33 (8.5)	14 (8.9)	19 (8.2)	
*Child–Pugh* (%)				0.88
A	299 (76.7)	121 (77.1)	178 (76.4)	
B	91 (23.3)	36 (22.9)	55 (23.6)	
*BCLC* (%)				**0.02**
0	123 (31.6)	60 (38.2)	63 (27.1)	
A	267 (68.4)	97 (61.8)	170 (72.9)	
*T.N.* (%)				0.17
Single	278 (71.3)	118 (75.2)	160 (68.7)	
Multiple	112 (28.7)	39 (24.8)	73 (31.3)	
*T.S.* (%)				**0.002**
< 3 cm	266 (68.2)	121 (77.1)	145 (62.2)	
≥ 3 cm	124 (31.8)	36 (22.9)	88 (37.8)	
AFP (ng/mL)	18.53 (4.45,178.73)	4.36 (2.74,8.47)	104.80 (21.71,427.90)	** < 0.001**
AFP-L3 (%)	0.00 (0.00,2.79)	0.00 (0.00,0.00)	1.00 (0.00,17.33)	** < 0.001**
DCP (ng/mL)	49.00 (26.75,229.50)	34.00 (22.50,59.50)	104.00 (31.00,478.00)	** < 0.001**
GALAD Score	2.75 (1.17,5.44)	0.79 (– 0.07,1.52)	4.97 (3.09,7.02)	** < 0.001**
WBC (10^9/L)	4.98 ± 1.89	4.97 ± 1.89	4.99 ± 1.90	0.94
RBC (10^12/L)	4.21 ± 0.62	4.26 ± 0.59	4.17 ± 0.64	0.14
Hb (g/L)	131.32 ± 19.69	132.03 ± 19.97	130.84 ± 19.53	0.56
ALT (U/L)	29.74 ± 16.92	28.23 ± 14.29	30.76 ± 18.45	0.15
AST (U/L)	30.02 ± 12.25	28.37 ± 10.41	31.13 ± 13.25	**0.02**
ALP (U/L)	89.69 ± 32.51	86.22 ± 30.00	92.02 ± 33.96	0.08
TBIL (μmol/L)	19.55 ± 10.06	19.95 ± 10.31	19.28 ± 9.90	0.52
DBIL (μmol/L)	7.97 ± 4.79	8.31 ± 5.22	7.74 ± 4.48	0.25
ALB (g/L)	37.12 ± 4.80	37.46 ± 4.77	36.90 ± 4.81	0.26
Glob (g/L)	28.88 ± 4.99	28.67 ± 4.79	29.03 ± 5.12	0.49
APTT (s)	33.38 ± 4.75	33.39 ± 3.99	33.36 ± 3.59	0.94
PT (s)	13.08 ± 4.09	13.17 ± 1.56	13.02 ± 1.45	0.36
TT (s)	15.19 ± 1.84	15.22 ± 1.83	15.17 ± 1.84	0.80
INR	1.17 ± 0.13	1.17 ± 0.14	1.16 ± 0.13	0.36

Continuous variables were expressed as mean ± standard deviation or medians (quartiles). Categorical variables were reported as frequency (percentage)

The bolded values indicate a P value less than 0.05, which represent statistical significance

*BCLC*, Barcelona Clinic Liver Cancer; *T.N*, tumor number; *T.S*, tumor size; *AFP,* alpha-fetoprotein; *AFP-L3*, lens culinaris agglutinin-reactive alpha-fetoprotein; *DCP*, des-gamma-carboxyprothrombin; *GALAD*, gender, age, AFP-L3, AFP, and DCP; *WBC*, leukocyte; *RBC*, red blood cell; *Hb*, hemoglobin; *ALT*, alanine aminotransferase; *AST*, aspartate aminotransferase; *ALP*, alkaline phosphatase; *TBIL*, total bilirubin; *DBIL*, direct bilirubin; *ALB*, albumin; *Glob*, globulin; *APTT*, activated partial thromboplastin time; *PT*, prothrombin time; *TT*, thrombin time; *INR*, international normalized ratio.

### The performance of GALAD score for predicting the recurrence

**Fig. 1:**
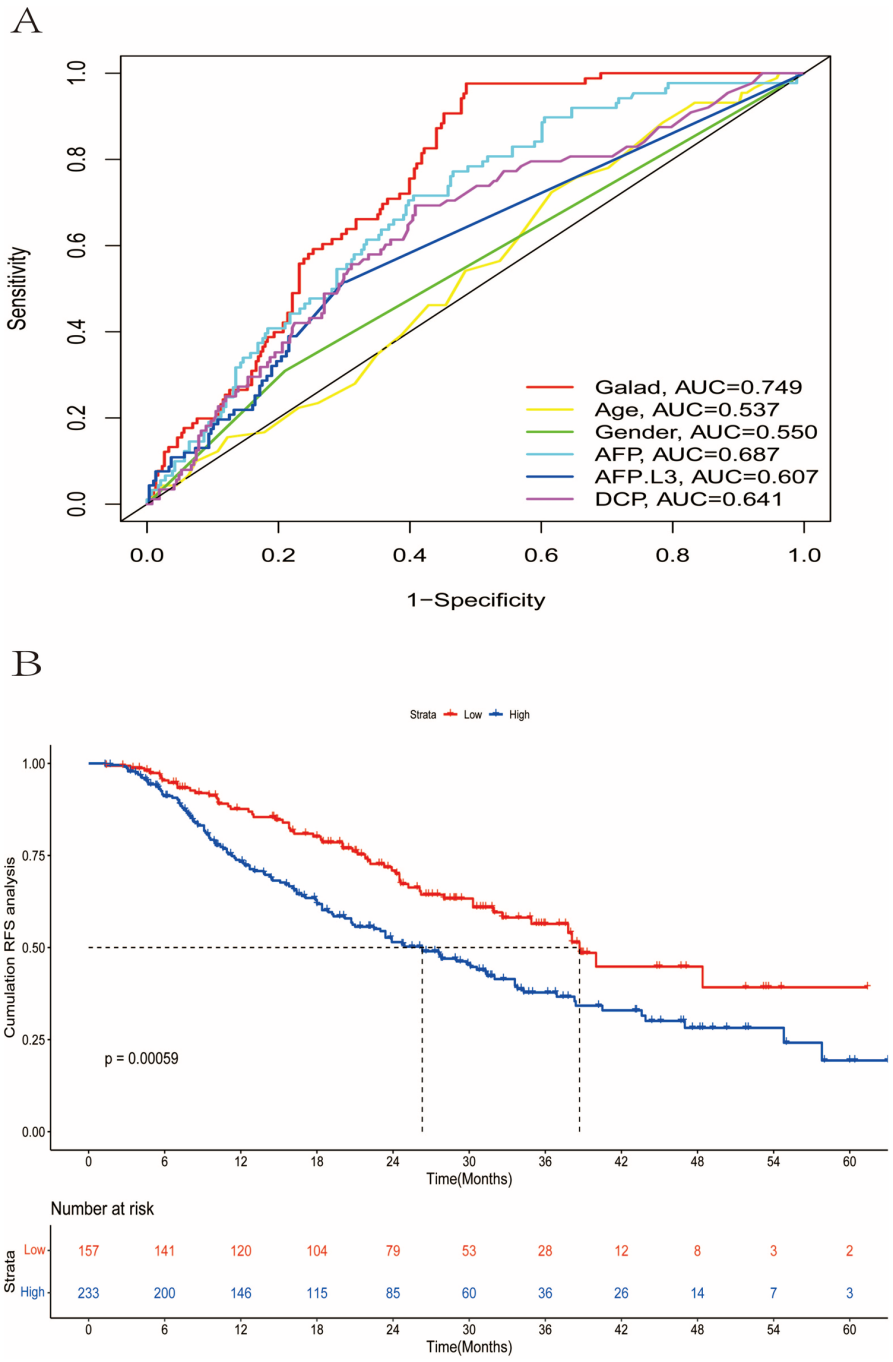
The predictive ability of GALAD score for recurrence of HCC. **A** The AUCs of GALAD, age, gender, AFP, AFP-L3, and DCP; **B** Kaplan–Meier curves of RFS for two groups classified by GALAD score. *AFP*, alpha-fetoprotein; *AFP-L3*, lens culinaris agglutinin-reactive alpha-fetoprotein; *DCP*, des-gamma-carboxyprothrombin; *GALAD*, gender, age, *AFP-L3*, AFP, and DCP; *HCC*, hepatocellular carcinoma; *AUC*, area under the receiver operating characteristic curve; *RFS*, recurrence-free survival

The performance of the GALAD score and other biomarkers for predicting the prognosis was evaluated by ROC curve (Fig. 1A). It revealed that AUCs were 0.537 for age, 0.550 for gender, 0.687 for AFP, 0.607 for AFP-L3, 0.641 for DCP, and 0.749 for the GALAD score, suggesting the favorable predictive ability of GALAD score. The optimum GALAD score cut-off for identifying the recurrence of HCC patients was 2.15, according to the Youden Index.

According to the cut-off value, patients could be classified into low-risk (*n* = 157) and high-risk (*n* = 233) groups. The comparison of baseline characteristics between two groups was shown in Table 1. There were significant differences between two groups in terms of the GALAD score and its related characteristics (age, gender, AFP, AFP-L3, and DCP). Of note, the high-risk group had more patients in BCLC stage A (72.9% vs. 61.8%) and had a higher prevalence of large tumors (37.8% vs. 22.9%). Furthermore, patients had higher AST levels in the high-risk group, indicating poor liver function.

The KM curve was plotted in Fig. 1B to compare the prognosis between two groups. The median RFS was 38.7 months (95% CI 33.6–43.8 months) in the low-risk group and 26.3 months (95% CI 21.0–31.6 months) in the high-risk group, respectively. The cumulative RFS rates for 1-, 3-, and 5-year were 87.6%, 56.4%, and 39.2% in the low-risk group, while 73.3%, 37.8%, and 19.3% in the high-risk group.

### Independent prognostic factors associated with RFS

**Fig. 2:**
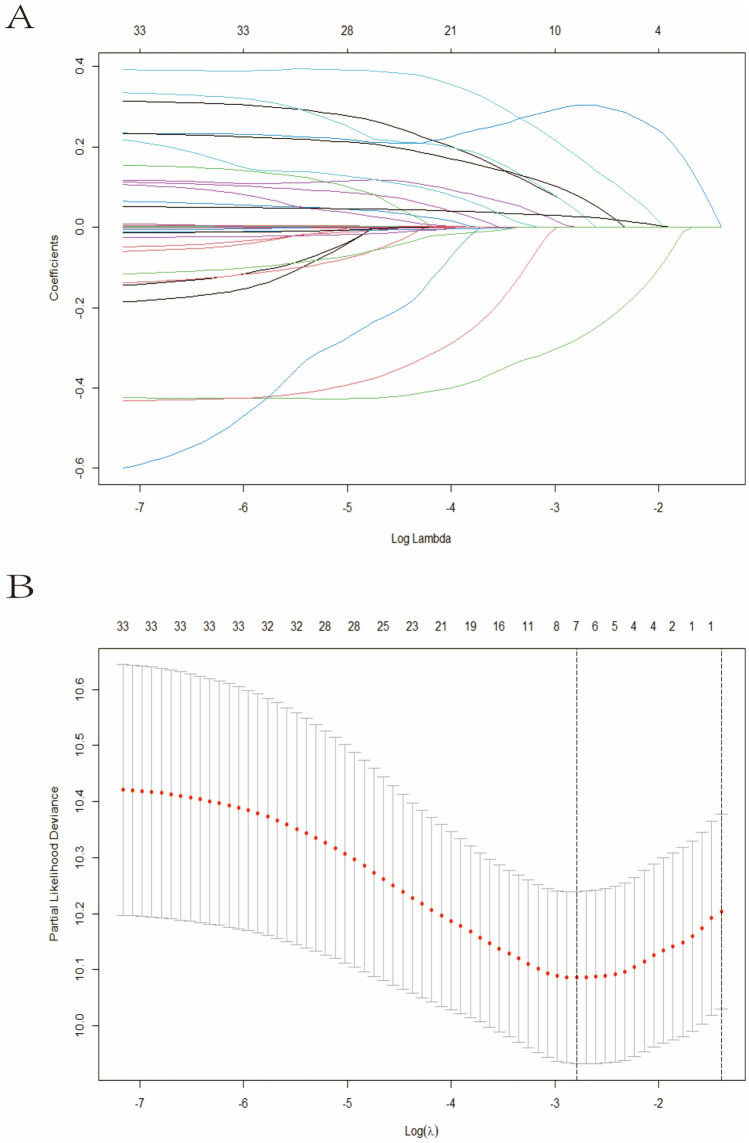
Selection of prognostic factors based on Lasso regression. **A** The variation characteristics of the coefficient of variables; **B** The selection process of the optimum parameter *λ* in the Lasso regression model by cross-validation method

Lasso regression analysis was employed to calculate the regression coefficient of prognostic factors for patients in high-risk group (Fig. 2A). According to Lasso regression with tenfold cross-validation, the optimal *λ* was 0.061 (Log *λ* = -1.212) (Fig. 2B). The factors included hypertension, antiviral, drinking, BCLC stage, tumor number, tumor size, and globulin. A Cox regression model was subsequently developed using parameters identified through Lasso regression, which contained tumor number (HR 1.485, 95% CI 1.015–2.411), tumor size (HR 1.136, 95% CI 1.051–1.519), and Globulin (HR 1.043, 95% CI 1.009–1.078) (Table 2).

**Table 2:** Multivariate Cox regression analysis based on the results of Lasso regression

Variables	HR (95%CI)	P value
Hypertension	1.256 (0.885–1.783)	0.203
Antiviral	0.663 (0.178–1.321)	0.514
Drinking	1.271 (0.898–1.798)	0.176
BCLC	1.261 (0.823–1.923)	0.287
T.N	1.485 (1.015–2.411)	**0.011**
T.S	1.136 (1.051–1.519)	**0.045**
Glob	1.043 (1.009–1.078)	**0.014**

The bolded values indicate a P value less than 0.05, which represent statistical significance

*BCLC*, Barcelona Clinic Liver Cancer; *T.N*, tumor number; *T.S*, tumor size; *Glob*, globulin

### Development of the nomogram

**Fig. 3:**
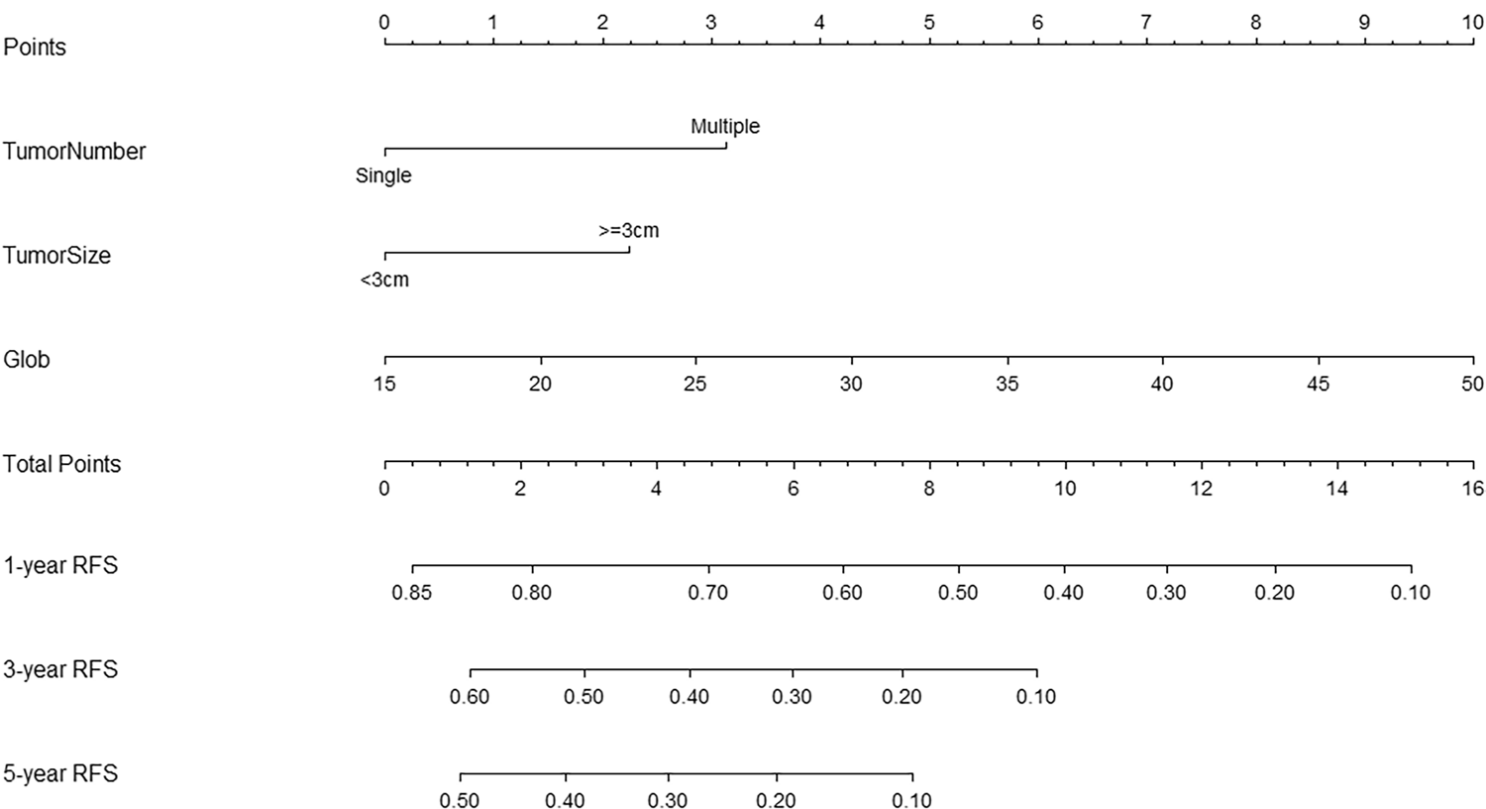
The nomogram, including tumor number, tumor size, and globulin, was used to predict time-related recurrence in high-risk patients

**Fig. 4:**
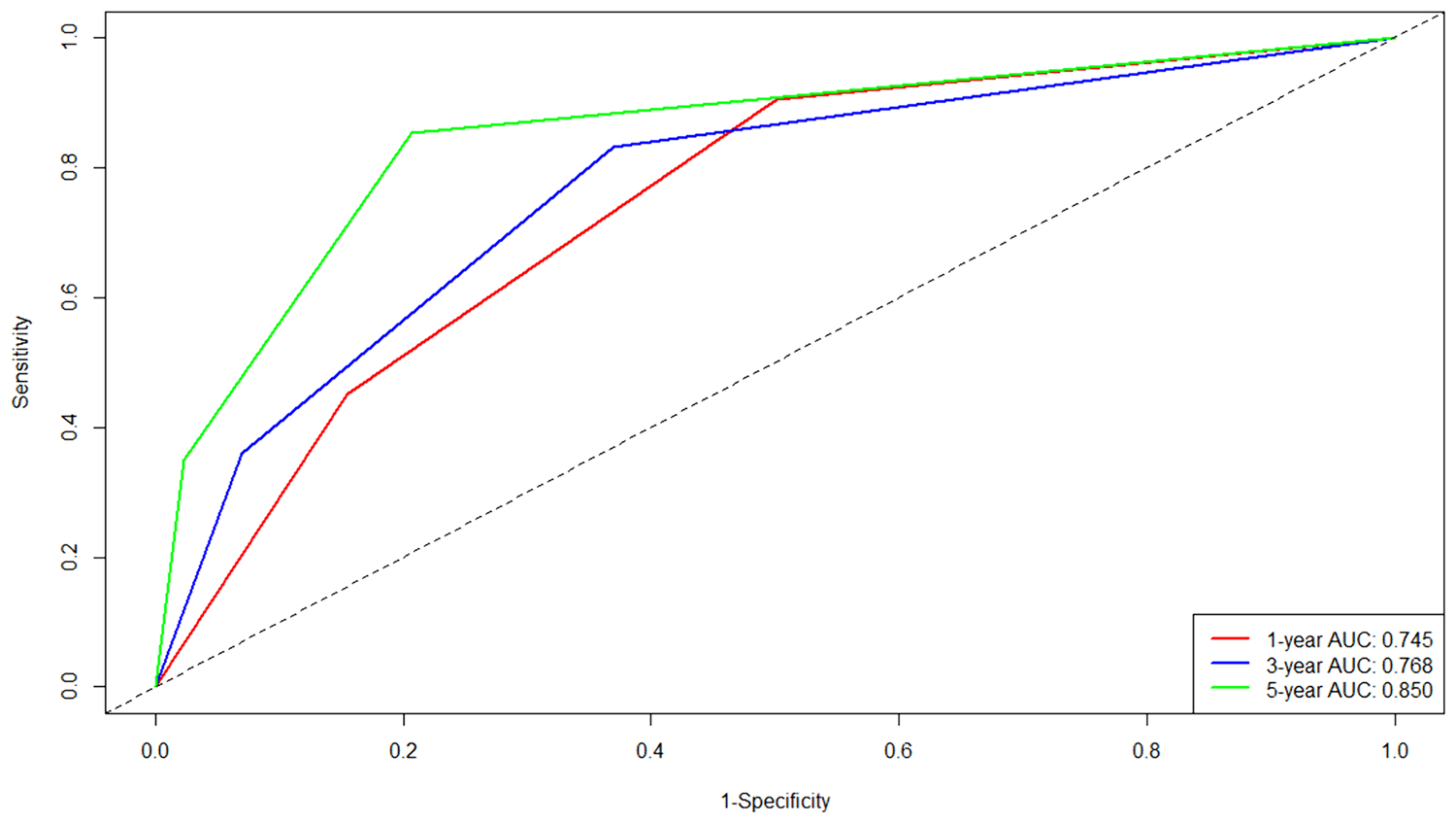
ROC curve of the nomogram. The AUCs of 1-, 3-, and 5-year were 0.745, 0.768, and 0.850, respectively. *ROC*, operating characteristic curve; *AUC*, area under the ROC curve

A prognostic nomogram integrating independent factors was developed (Fig. 3). The AUCs of 1-, 3-, and 5-year, as shown by time-dependent ROC curves, were 0.745, 0.768, and 0.850, respectively (Fig. 4). These outcomes indicated the nomogram's advantageous diagnostic efficacy. The calibration curves (Fig. S1) and decision curve analysis (DCA) curves (Figure S2) were further used, which revealed reliable calibration and clinical applicability of the nomogram.

**Fig. 5:**
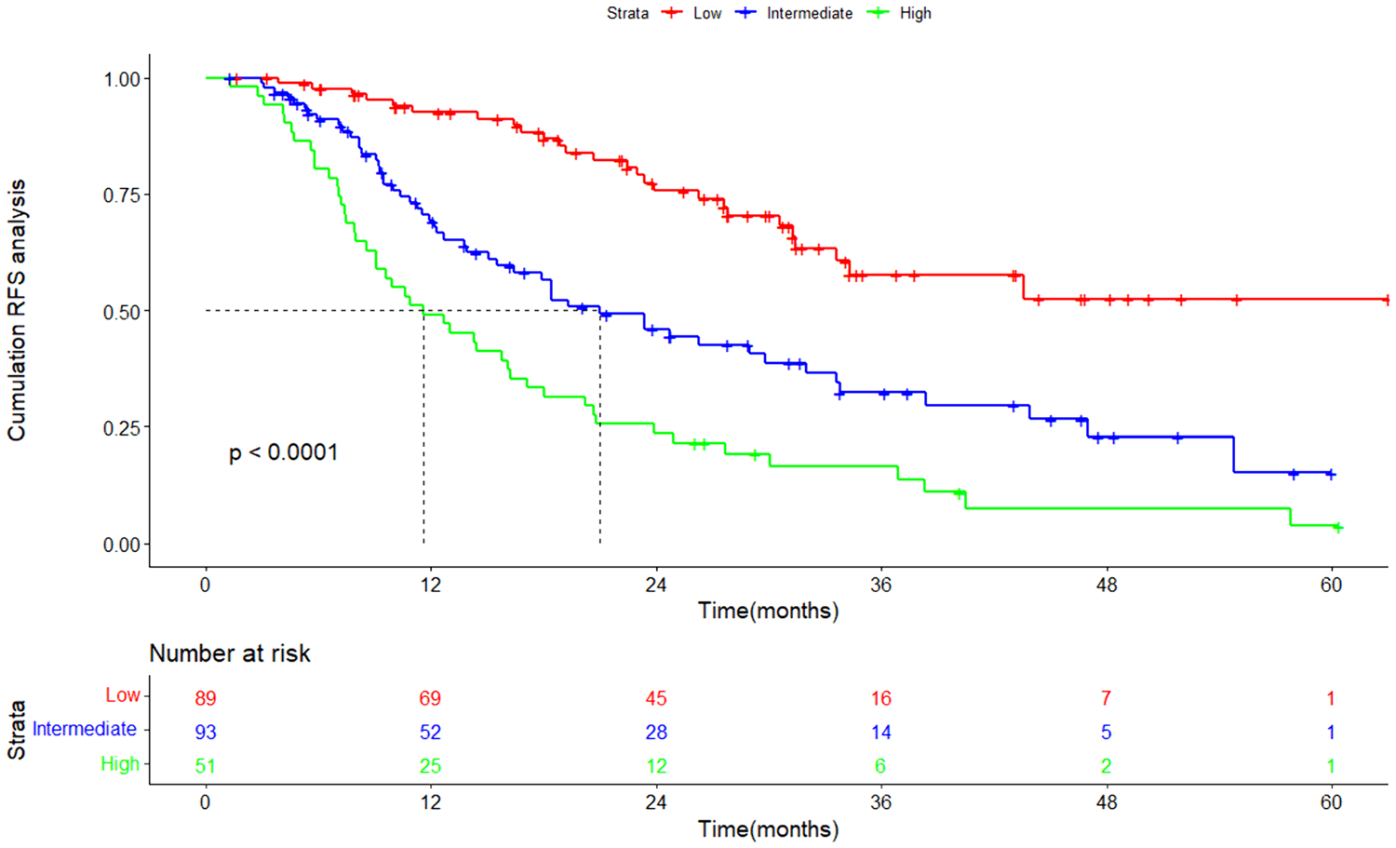
Kaplan–Meier curves of RFS for low-risk group, intermediate-risk group, and high-risk group classified by nomogram. *RFS*, recurrence-free survival

Based on the nomogram, patients were categorized into low-risk (*n* = 89), intermediate-risk (*n* = 93), and high-risk (*n* = 51) groups. The KM curve was then plotted, revealing that the median RFS was 21.0 months for the intermediate-risk group and 11.6 months for the high-risk group, while it was not reached in the low-risk group (Fig. 5). The cumulative RFS rates for 1-, 3-, and 5-year were 92.5%, 57.7%, and 52.4% in the low-risk group, 69.2%, 32.2%, and 15.2% in the intermediate-risk group, whereas 49.0%, 16.4%, and 3.7% in the high-risk group.

## Discussion

Primary liver cancer, particularly HCC, is a substantial public health problem and a leading cause of death globally (Ganesan and Kulik 2023; Forner et al. 2018). At present, the high recurrence rate of HCC continues to affect patients' quality of life, emphasizing the importance of developing more accurate surveillance tests (Nevola et al. 2023). Therefore, our retrospective cohort study included 390 HCC patients after local ablation and assessed the performance of GALAD score for forecasting the prognosis. The results showed that the GALAD score is an excellent biomarker model, exhibiting good predictive power in RFS. Moreover, we established a nomogram based on the independent prognostic factors in high-risk patients, aiming to help guide clinical decision-making.

Local ablation, which is considered as the first-line treatment for early-stage HCC, is widely used in clinical applications. During ablation therapies, an ablation needle is inserted externally into the tumor tissue, and the heat produced around the needle is utilized to damage tumor cells (Chen et al. 2022; Breen and Lencioni 2015). Long-term clinical practice has confirmed that local ablation could effectively prolong the overall survival and recurrence-free survival of HCC patients (Xu et al. 2017). Thus, we adopted local ablation in our research and predicted the postoperative recurrence of patients with HCC.

The GALAD score served as a diagnostic model, which was developed based on data from 670 patients with either chronic liver disease alone or HCC in the United Kingdom. In this cohort, the model gave values of more than 0.88 when optimized for sensitivity and specificity (Johnson et al. 2014). Numerous studies have confirmed that the GALAD demonstrates exceptional ability in the early diagnosis of HCC these years. A multicenter prospective study enrolled 1561 eligible participants and validated the effectiveness of GALAD score for early diagnosis of HCC in Chinese patients. The GALAD score was capable of detecting HCC at 24 weeks (AUC: 0.848) and even 48 weeks (AUC: 0.833) across the population (Huang et al. 2022). Similarly, GALAD demonstrated high diagnostic accuracy in a multinational study including 6834 patients from Asian and European (Berhane et al. 2016).

However, to our knowledge, few researches focused on the ability of GALAD score for predicting the prognosis currently. A study by Cagnin et al. included 212 patients and observed that the high GALAD level was associated with decreased survival (Cagnin et al. 2023). In our research, we concentrated on the performance of GALAD score to predict the recurrence in HCC patients who underwent ablation. The ROC curve (AUC: 0.749) demonstrated better predictive capability of GALAD score compared to age, gender, AFP, AFP-L3, and DCP. Meanwhile, GALAD score could stratify HCC patients into two distinct groups with low-risk or high-risk of recurrence. These outcomes provided clinical evidence of the prognostic ability of the GALAD for HCC patients after local ablation.

Clinical characteristics in the GALAD model include age, gender, AFP, AFP-L3, and DCP. Within these characteristics, age and gender are recognized risk factors for prognosis in HCC patients. Elderly patients with HCC frequently exhibit poor liver function and fast tumor progression, leading to a high recurrence rate (Chu and Chok 2019). As for male HCC patients, the prognosis is also unsatisfactory due to reasons such as androgen and lifestyle (Bashir Hamidu et al. 2021; Ao et al. 2012). AFP, a major plasma protein produced by liver cells, has been widely applied as a diagnostic and prognostic predictor in patients with HCC for decades (Galle et al. 2019; Hu et al. 2022). Some studies proposed that AFP may regulate the growth of tumor cells by mechanisms, including apoptotic regulation and cytoplasmic signaling modulation (Lin et al. 2017). AFP-L3 is the core-fucosylated N-glycoform of AFP and has a more specific detection and surveillance value in HCC compared to AFP (Toyoda et al. 2011). In addition, DCP is an atypical form of prothrombin that lacks posttranslational modification with gamma-carboxylation (Y. Yang et al. 2021). The high level of DCP is reportedly associated with portal vein invasion and poor prognosis (Koike et al. 2001). A study by Norman et al. found that the combination of AFP-L3 and DCP strongly predicted HCC recurrence after liver transplantation. In this study, the recurrence-free survival rate was 43.7% for patients with dual-positive biomarkers compared to 97.0% for others at three years (Norman et al. 2023).

Notably, the median RFS was only 26.3 months (95% CI 21.0–31.6 months) in the high-risk group classified by GALAD score. Due to the unfavorable prognosis of these patients, further surveillance is required. Consequently, Lasso-Cox regression was used to screen independent prognostic factors and establish a nomogram in our study (Deo 2015). According to the nomogram (tumor size, tumor number, and globulin), high-risk patients classified by GALAD score were further categorized into different groups, which could help clinicians to accurately identify those at risk of recurrence and realize precision medicine. Tumor burden, including tumor size and tumor number, is commonly viewed as the key characteristic of cancer. There is plenty of evidence indicating that tumor burden holds prognostic value in a variety of human neoplasms (Kim et al. 2020; Wang et al. 2022). Globulin is a class of immune proteins, and its levels could reflect the synthetic function of the liver (Perez et al. 2017). As a liver function parameter, globulin has been confirmed to have prognostic value for HCC patients in several studies (Zhang et al. 2019; Deng et al. 2016).

Inevitably, there are some limitations in our research. Firstly, as a retrospective study, this study involves a certain degree of bias. Additionally, our study was a single-center research, and the sample size was insufficient. Thus, we didn’t develop the nomogram for patients in low-risk group. Finally, in our cohort, there were only the patients who received local ablation. More studies are required to investigate the application of our outcomes in patients undergoing different treatments, such as surgery or liver transplantation.

## Conclusion

In summary, our study indicated that the GALAD score is a potentially useful predictive tool for the recurrence of HCC. In order to further surveillance, we developed a nomogram for high-risk patients classified by GALAD score. The nomogram, including tumor size, tumor number, and globulin, demonstrated excellent predictive ability.

### Supplementary Information

Below is the link to the electronic supplementary material.

Fig. S1. Calibration curve for predicting 1-, 3-, and 5-year RFS. RFS, recurrence-free survival (TIFF 2450 kb)

Fig. S2. DCA for 1-, 3-, and 5-year RFS. (A) DCA for 1-year RFS; (B) DCA for 3-year RFS; (C) DCA for 5-year RFS. DCA, decision curve analysis; RFS, recurrence-free survival (JPG 1051 kb)

## Data Availability

All relevant data are available within the manuscript and its supplementary material files. Further inquiries can be directed to the corresponding author (Caixia Hu, hucaixia1217@126.com).
