# Supplementary figures and images for: GALAD score as a prognostic model for recurrence of hepatocellular carcinoma after local ablation
Source: J Cancer Res Clin Oncol. 2024 May 7;150(5):241. doi: 10.1007/s00432-024-05760-z (PMC11076334; doi:10.1007/s00432-024-05760-z)

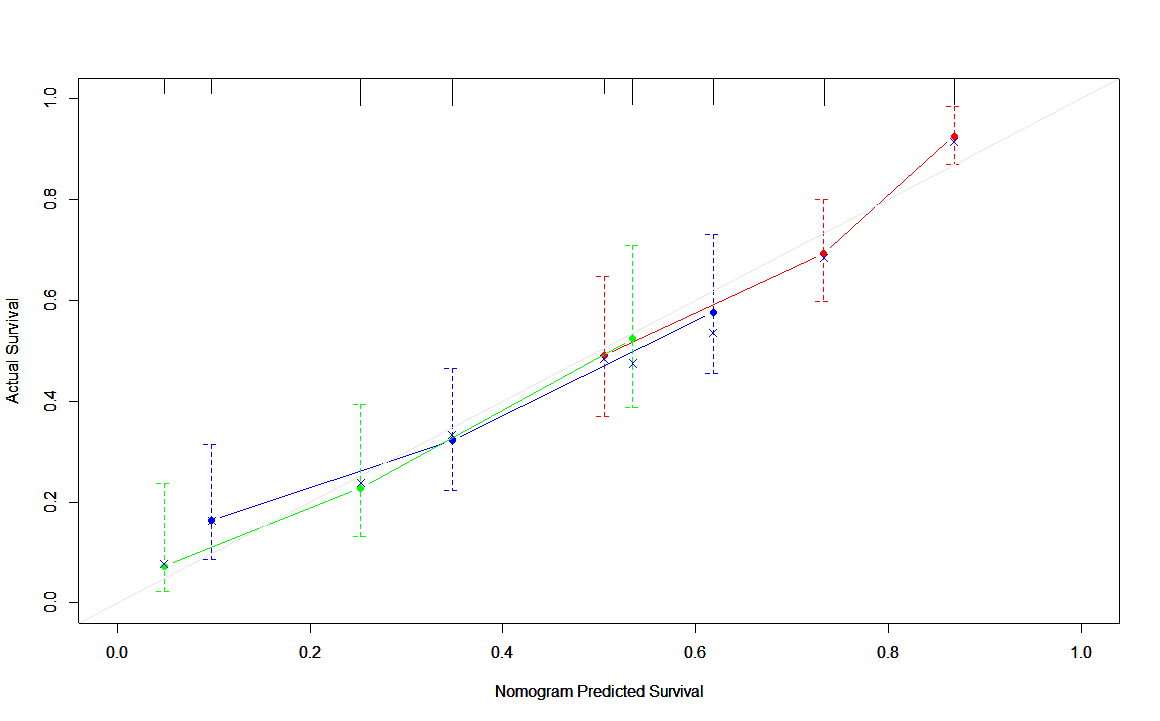

Supplement: Supplementary file 1 — Fig. S1. Calibration curve for predicting 1-, 3-, and 5-year RFS. RFS, recurrence-free survival (TIFF 2450 kb) [file 432_2024_5760_MOESM1_ESM.tiff]

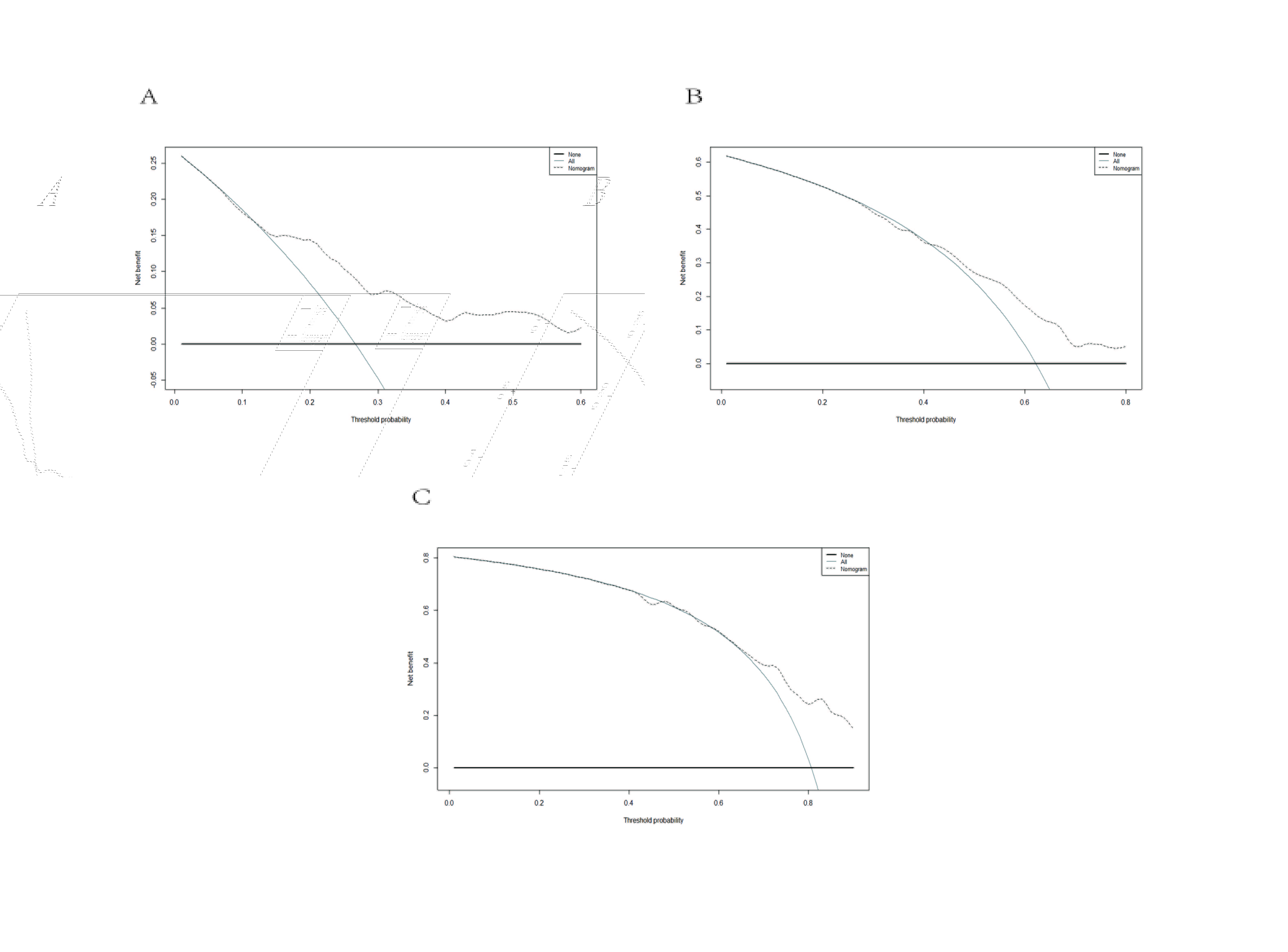

Supplement: Supplementary file 2 — Fig. S2. DCA for 1-, 3-, and 5-year RFS. (A) DCA for 1-year RFS; (B) DCA for 3-year RFS; (C) DCA for 5-year RFS. DCA, decision curve analysis; RFS, recurrence-free survival (JPG 1051 kb) [file 432_2024_5760_MOESM2_ESM.jpg]
